# 原发性肺肉瘤19例临床分析

**DOI:** 10.3779/j.issn.1009-3419.2012.06.09

**Published:** 2012-06-20

**Authors:** 坤 刘, 为民 李

**Affiliations:** 610041 成都，四川大学华西医院呼吸内科 Department of Respiratory Medicine, West China Hospital, Sichuan University, Chengdu 610041, China

**Keywords:** 原发性肺肉瘤, 诊断, 治疗, Primary pulmonary sarcoma, Diagnosis, Treatment

## Abstract

**背景与目的:**

原发性肺肉瘤（primary pulmonary sarcoma, PPS）是一种极少见的肺部原发性恶性肿瘤，起源于肺间叶组织，极易误诊，本研究旨在探讨原发性肺肉瘤的临床特点，以期为临床早期诊断及治疗提供更全面的参考依据。

**方法:**

分析总结四川大学华西医院1996年-2011年经病理证实的19例PPS患者的临床资料并进行文献复习。

**结果:**

19例患者中男性12例，41岁-68岁患者占57.9%，平均年龄41岁。主要症状为咳嗽、咳痰以及咯血；影像学表现中8例见“毛刺征”，5例见“分叶征”，主要病理学类型为滑膜肉瘤、平滑肌肉瘤以及恶性纤维组织细胞瘤。手术治疗17例，化疗1例。随访17例，随访率89.5%，中位生存时间为18个月。

**结论:**

原发性肺肉瘤临床及影像学均无特征性表现，易被误诊，死亡率高。

## Abstract (en)

**Background and objective:**

Primary pulmonary sarcoma (PPS) is a rare malignant tumor that originates from the mesenchymal tissue of the lungs. PPS is often easily misdiagnosed. This study aims to explore the clinical characteristics of PPS and provide a more comprehensive reference for the early diagnosis and treatment of this disease.

**Methods:**

The 1996 to 2011 clinical data of 19 cases with PPS in West China Hospital were presented, along with literature review.

**Results:**

A total of 19 patients, which include 12 males, were presented in this study. Patients with age between 41 and 68 years accounted for 57.9% (the mean age was 41 years). The main clinical manifestations were cough, expectoration, and hemoptysis. Based on the imaging results, spiculation was found in eight cases and lobulation in five cases. The main pathologic types were synovial sarcoma, leiomyosarcoma, and malignant fibrous histiocytoma. Seventeen patients underwent surgery, whereas one patient underwent chemotherapy. All 17 cases with a median survival of 18 months were investigated after being discharged (follow-up rate: 89.5%).

**Conclusion:**

The clinical and imaging features of PPS are not characteristic. Thus, PPS is easily misdiagnosed and can cause high mortality among patients.

原发性肺肉瘤（primary pulmonary sarcoma, PPS）非常少见，国外文献报告PPS占肺部原发性恶性肿瘤的1%-4%^[1, 2]^，国内报告为0.7%-3.6%^[3, 4]^，多数为个案报告。本研究分析四川大学华西医院1996年-2011年经病理证实的19例PPS患者的临床资料并进行文献复习，以加深对本病的了解，为临床早期诊断、治疗提供更全面的参考依据。

## 1 临床资料

1996年-2011年四川大学华西医院共有19例患者经病理确诊为PPS，其中男性12例，女性7例，性别比1.7:1；年龄1岁-68岁，其中41岁-68岁者占57.9%，平均年龄41岁。所有患者均进行了临床症状、影像学表现及病理学检查的总结，包括性别、年龄、临床症状、入院诊断、出院诊断、肿瘤部位、形状、大小、肿瘤边缘情况、内部是否有空洞、钙化、增强扫描是否强化、有无胸腔积液以及其病理组织学、免疫组化和治疗方法，同时对其中17例进行了随访。

## 2 结果

### 2.1 临床症状

主要症状包括咳嗽、咳痰、咯血、发热、胸痛、呼吸困难，其中咳嗽13例（68.4%）、咳痰10例（52.6%）、咯血7例（36.8%）、发热3例（15.8%）、胸痛5例（26.3%）、呼吸困难8例（42.1%），2例患者无明显临床症状，均为体检时影像学检查发现肿瘤（10.5%），1例为声音嘶哑（5.3%）。在治疗过程中所有患者的症状都随着病情的发展而变化，并非固定不变。

### 2.2 影像学表现

19例患者均行胸部CT增强扫描。影像学检查（表 1）示病变部位：左肺12例（63.2%），右肺7例（36.8%）；病灶形状：17例为包块影，2例为斑片影；病灶大小：肿瘤直径≤5 cm者5例（26.3%），肿瘤直径 > 5 cm且≤10 cm者8例（42.1%），4例患者肿瘤巨大（肿瘤直径 > 10 cm）占21.1%；病灶边缘：6例病灶边缘清楚（31.6%），13例病灶边缘模糊（68.4%），其中8例见毛刺征（42.1%）；4例病灶内部可见空洞（21.1%），2例病灶可见钙化（10.5%）；6例患者有胸腔积液（31.6%）；CT增强扫描：9例患者其病灶有强化（47.4%），均为病灶不均匀强化。

**1:** 患者基本情况及影像学表现 Basic information and imaging findings of the patients

No.	Gender	Age	Location	Shape	Size (cm)	Border	Cavity or calcification	Enhancement	Pleural effusion
1	Female	21	R-Hilum	Lobulation	5.3×4.8	Well defined	-	Yes	R-side
2	Male	62	L-Hilum	Irregular	9.2×6.4	Spiculation	-	Yes	-
3	Female	34	LLL	Round	Φ2	Spiculation	-	-	-
4	Male	51	LULL	Lobulation	11.8×7.4	Well defined	Cavity & Calcification	Yes	L-side
5	Male	42	RUL	Round	Φ3.9	Well defined	Calcification	-	-
6	Male	54	LUL	Lobulation	3.5×3.1	Spiculation	-	Yes	-
7	Male	37	RUL	Plaque & Stripe	-	Unsharpness	-	-	-
8	Male	68	LUL	Lobulation	7.8×6.5	Spiculation	-	-	-
9	Female	53	LUL	Lobulation	6×5	Well defined	Cavity	Yes	-
10	Female	3	RUMLL	Irregular	Occupy the whole cavity	Spiculation	Cavity	-	R-side
11	Male	55	LLL	Plaque	-	Unsharpness	-	-	-
12	Female	1	LULL	Irregular	Occupy the whole cavity	Spiculation	-	Yes	L-side
13	Male	27	LULL	Irregular	15×13	Spiculation	-	Yes	L-side
14	Female	42	RUL	Irregular	16×14	Unsharpness	-	Yes	-
15	Male	68	L-Hilum	Irregular	5.0×7.5	Unsharpness	-	-	Bilateral
16	Male	63	LUL	Round	Φ8 cm	Spiculation	-	Yes	-
17	Male	48	LUL	Stripe	6×11	Well defined	Cavity	-	-
18	Female	28	RUL	Round	Φ4	Well defined	-	-	-
19	Female	30	RLL	Irregular	3×5	Unsharpness	-	-	-
R-Hilum: hilum of right lung; L-Hilum: hilum of left lung; LLL: left lower lobe; LULL: left upper and lower lobes; RUL: right upper lobe; LUL: left upper lobe; RUMLL: right upper, middle and lower lobes; RLL: right lower lobe; Φ: diameter; R-side: right-side; L- side: left-side									

### 2.3 病理学类型

经病理学检查证实19例原发性肺肉瘤中滑膜肉瘤5例（26.3%），平滑肌肉瘤4例（21.1%），恶性纤维组织细胞瘤3例（15.8%），胸膜肺母细胞瘤2例（10.5%），横纹肌肉瘤2例（10.5%），纤维肉瘤1例（5.3%），腺泡状软组织肉瘤1例（5.3%），软骨肉瘤1例（5.3%）。

### 2.4 免疫组化检测

本研究中19例患者有18例行免疫组化染色，另有1例肺软骨肉瘤未做免疫组化直接行石蜡切片以进行组织学诊断。18例患者的免疫组化结果显示，细胞角蛋白（cytokeratin, CK）（-）11例/16例，阴性率为68.8%；上皮膜抗原（epithelial membrane antigen, EMA）（-）9例/13例，阴性率为69.2%；波形蛋白（vimentin, Vim）（+）11例/11例，阳性率为100%；结蛋白（desmin, Des）（+）7例/16例，阳性率为43.8%；S-100蛋白（S-100 protein, S-100）（-）16例/17例，阴性率为94.1%；平滑肌抗原（smooth muscle antigen, SMA）（-）8例/11例，阴性率为72.7%。

5例滑膜肉瘤中Vim阳性率100%（3/3），EMA阳性率、CK阳性率、S-100阴性率均为80%（4/5），Des阴性率和CD99阳性率为100%（4/4），2例检出SS18基因易位。

4例平滑肌肉瘤中Vim阳性率（2/2）、Des阳性率（3/3）、肌特异性肌动蛋白（muscle specific actin, MSA）阳性率（2/2）、SMA阳性率（3/3）、S-100阴性率（4/4）均为100%，CK阳性率为50%（1/2）。

3例恶性纤维组织细胞瘤中Vim阳性率、EMA阴性率、CK阴性率、S-100阴性率、Des阴性率100%、SMA阴性率均为100%（3/3）。

2例胸膜肺母细胞瘤中Vim阳性率100%（1/1），EMA阴性率、CK阴性率、Des阳性率均为100%（2/2）；肌细胞生成素（Myogenin）1例为（+），1例为部分（+）。

2例横纹肌肉瘤中Des阳性率、Myogenin阳性率、CK阴性率均为100%（2/2）。

1例纤维肉瘤中Vim（+）、S100（-）、SMA（-）、Des（-）、CD34（-）、EMA（-）、CD56（+）、CK（-）、CD5（-）、CD23（-）、神经元特异性烯醇化酶（neuron-specific enolase, NSE）（-）、CD20（-）。

1例腺泡状软组织肉瘤中Vim（+）、Des（-）、Myogenin（-）、CK（-）、EMA（-）、S-100（-）、CD68（-）、NSE（+）。

### 2.5 入院诊断

入院时仅有1例患者在门诊经纤维支气管镜病检明确诊断，15例患者入院诊断均怀疑是肺癌（占78.9%），1例患者诊断为肺脓肿，1例患者诊断为肺大疱，1例患者诊断为结核性胸膜炎。

### 2.6 治疗

17例患者行手术治疗（89.4%），其中11例行淋巴结清扫术，除1例因肿瘤太大仅行部分切除术，其余均为根治性手术。术后4例患者接受化疗，1例接受放疗；1例行支气管动脉化疗药物灌注术；1例经皮肺穿刺病理学检查确诊后放弃治疗要求出院。

### 2.7 随访

19例原发性肺肉瘤患者中随访17例，失访2例，随访率89.5%（表 2）。17例患者中中位生存时间为18个月。除1例目前术后生存7个月，其余16例均死亡：死于呼吸衰竭6例，死于严重咯血1例，死于远处脏器转移3例（1例全身转移，1例脑转移，1例左侧肾上腺转移），死于胸内转移4例（2例胸膜转移，2例对侧肺部转移），死于肿瘤复发2例（手术切除并且术后症状改善2个月以上在支气管残端又发现性质相同的肿瘤）。生存期≤1年者7例（41.2%），生存期≤3年者12例（70.6%），生存期≤5年者14例（82.4%），生存期超过5年者2例（11.8%）。

**2:** 患者的临床症状、病理诊断、治疗方式及生存期 Clinical characteristics, pathology diagnosis, treatment and survival time of the patients

No.	Co-ugh	Expecto-ration	Hemo-ptysis	Fe-ver	Chest pain	Dys-pnea	Other symptoms	Pathologic diagnosis	Treatment	Survival time (months)
1	Yes	Yes	Yes	Yes	Yes	-	-	Alveolar soft part sarcoma	Surgery	> 7
2	Yes	-	-	-	-	-	-	Synovial sarcoma	Chemotherapy	6
3	Yes	Yes	Yes	-	-	-	-	Synovial sarcoma	Surgery & Chemotherapy	5
4	Yes	Yes	Yes	-	Yes	-	-	Fibrosarcoma	Surgery & Chemotherapy	18
5	-	-	-	-	-	-	Found by health examination	Chondrosarcoma	Surgery	34
6	Yes	Yes	Yes	-	-	-	-	Malignant fibrous histiocytoma	Surgery	1
7	-	-	-	-	-	Yes	-	Synovial sarcoma	Surgery	33
8	Yes	Yes	-	-	-	-	-	Rhabdomyosarcoma	Surgery	3
9	Yes	Yes	Yes		-	-	-	Synovial sarcoma	Surgery	4
10	Yes	Yes	-	Yes	-	-	-	Pleuropulmonary blastoma	Surgery	1
11	Yes	Yes	-	-	-	Yes	-	Malignant fibrous histiocytoma	Surgery	62
12	-	-	-	-	-	Yes	-	Pleuropulmnary blastoma	Surgery	Not available
13	Yes	Yes	Yes	-	Yes	Yes	-	Synovial sarcoma	Not Known	Not available
14	Yes	-	-	-	Yes	Yes	-	Leiomyosarcoma	Surgery & Chemotherapy	9
15	Yes	-	-	Yes	-	Yes	-	Leiomyosarcoma	Surgery & Chemotherapy	21
16	-	-	-	-	-	Yes	-	Leiomyosarcoma	Surgery & Chemotherapy	21
17	Yes	Yes	Yes	-	-	-	-	Malignant fibrous histiocytoma	Surgery	27
18	-	-	-	-	-	-	Found by health examination	Rhabdomyosarcoma	Surgery	50
19	-	-	-	-	Yes	Yes	-	Leiomyosarcoma	Surgery	43

## 3 讨论

PPS是一种极少见的恶性肿瘤，起源于肺实质、支气管壁、血管壁和支气管软骨等处的间叶组织，可发生于任何年龄，好发于17岁-67岁，尤其以41岁-46岁居多，多见于青壮年，发病年龄较原发性支气管肺癌年轻，易误诊为肺癌或纵隔良性疾病。男性多于女性，但两性发病率差异不如肺癌明显，文献报告为1.2:1-1.7:1^[2]^。本研究中19例PPS患者平均发病年龄41.4岁，男女比为1.7:1，与文献一致。

从组织学上通常将PPS归类为恶性纤维组织细胞瘤、平滑肌肉瘤、横纹肌肉瘤、滑膜肉瘤和纤维肉瘤等。文献^[1, 5]^报道最多的是平滑肌肉瘤，其次是恶性纤维组织细胞瘤。2004年WHO^[6]^将PPS分为血管肉瘤、胸膜肺母细胞瘤、滑膜肉瘤、肺动脉肉瘤、肺静脉肉瘤等。本研究中滑膜肉瘤最多见，其次为平滑肌肉瘤和恶性纤维组织细胞瘤，与文献报道不一致。

PPS的临床症状与肿瘤的性质、部位、大小及生长速度有关，主要临床症状为咳嗽、痰中带血及胸痛，部分患者无症状。本研究中2例患者无明显临床症状，为体检时影像学检查发现肿瘤，均为周围型肿瘤且直径 < 5 cm，与文献相符。周围型PPS患者早期多无明显症状，出现症状时肿瘤多已很大，而中央型肿瘤患者症状出现较早。当肿瘤增大压迫或侵袭支气管，则会引起严重的咳嗽、咳痰或咯血，肿瘤累及胸膜时可出现胸痛。本研究中肿瘤累及肺门区的患者其临床症状均较周围型的患者明显，但差异无统计学意义（*P*=0.13），可能因病例数较小，且临床症状同时受肿瘤性质、大小等因素影响所致。

PPS通常首先由影像学检查所发现，其胸部影像学检查多表现为边缘光滑的分叶状类圆形肿块影，密度多均匀，少数病例有毛刺征，肿瘤呈膨胀性生长，偶有跨叶生长，可侵袭周围组织。PPS以周围型为主，直径较大，一般 > 5 cm，甚至可占据整个肺叶^[7]^。本研究中2例胸膜肺母细胞瘤患者，其病灶几乎占据一侧胸腔。肺肉瘤表面常有包膜或假包膜形成，肿块中央常见低密度坏死灶，多可见钙化^[8]^。增强扫描后病变多呈不均匀强化，肺癌常表现瘤体均匀强化或瘤体内点线状、斑片状强化，据此可作鉴别。PPS极少累及淋巴结，肿瘤靠近胸膜者，常累及脏层胸膜，出现胸腔积液^[9]^。中央型PPS影像学表现缺乏特征性，表现为肺门区支气管内结节或周围肿块，向腔内外生长，生长缓慢，常有阻塞性炎症及阻塞性肺不张，易与肺癌混淆^[10]^。本研究中11例为周围型（57.9%），8例为中央型（42.1%），9例患者CT增强扫描病灶呈现不均匀强化，瘤周多可见到不规则厚片状或环形强化，中央区域强化不明显，出现液化坏死者可见低密度影，出现钙化灶则呈高密度影。

PPS的确诊主要依靠细胞形态学和免疫组织化学。PPS很少侵犯支气管上皮，故痰脱落细胞学检查阳性率较低。中央型肺肉瘤经纤维支气管镜多可见支气管受压、变窄，其内可见新生物，触之易出血，但纤维支气管镜活检通常因取得组织较少而诊断困难。经皮肺穿刺适用于周围型肺肉瘤，但也常因取得坏死组织或组织较少细胞学检查易被误诊为肺癌。因PPS肺门及纵隔淋巴结转移少见，故电视纵隔镜检查意义不大。Janssen等^[2]^认为胸腔镜手术除可取活检外还可观察肿瘤对周围组织的浸润，对诊断有帮助且可避免诊断性开胸手术。PPS的确诊依赖病理学检查。PPS实质呈鱼肉状，切面常见出血、坏死。本研究中手术切除的PPS肿瘤组织大多质脆，触之易出血，剖开包块，切面似鱼肉样，部分肿瘤内部有大量坏死组织，呈豆渣样，与文献相符。由于在光镜下PPS易与肉瘤样癌、癌肉瘤相混淆，因此相关标记物的免疫组化染色，有助于排除上皮来源可能及间叶成分的确定^[11, 12]^。肺肉瘤免疫组化一般表现为Vim（+）、Des（+）、CK（-）、EMA（-）、S-100蛋白（-）。Vim存在于几乎所有的间叶细胞中，是一种良好的间叶来源肿瘤标记物，多用于肉瘤与癌的鉴别，与CK一起应用可以区分绝大多数上皮和间叶来源的肿瘤；Des是肌源性分化的特异性标记物；CK和EMA是上皮相关标记物，但在滑膜肉瘤中也有表达；SS18基因易位是滑膜肉瘤的特异性分子标记^[13]^。本研究中Vim阳性率100%，Des阳性率43.8%（肌源性肺肉瘤中Des阳性率100%），CK阴性率68.8%，EMA阴性率69.2%，S-100阴性率94.1%，与文献相符。

PPS呈膨胀性生长，多与周围组织分界清楚，表面常有包膜或假包膜形成，因此手术切除率较高，首选根治性手术，并根据具体情况行淋巴结清扫术、胸导管结扎术及胸膜粘连松解术。相对手术禁忌症为：①癌症晚期，全身广泛转移，多器官功能衰竭者；②凝血功能障碍、有严重出血倾向者；③全身一般情况差，不能耐受手术者。经完全切除者生存率明显高于放疗和未经治疗者^[12]^，但在手术方式的选上择尚存分歧。Trassard等^[14]^认为PPS局部切除或肺段切除同根治性切除效果相同，但一般因肿瘤较大不宜行局部楔形切除或肺段切除。Halyard等^[15]^认为本病有局部复发倾向，行根治性切除更为可靠。PPS淋巴结转移较少见，对于是否进行区域淋巴结清扫应根据具体情况进行判断。Bacha等^[16]^对3例PPS侵犯肺动、静脉或心房者，在体外循环下切除肿瘤取得了较好效果。亦有报道^[17]^对部分恶性程度较高的肺肉瘤进行术前化疗。一般认为放疗对PPS有一定的治疗价值，化疗效果不理想。高度恶性的肺肉瘤预后不良，可尝试给予辅助治疗。有明显外侵等无法彻底切除并有淋巴结转移的病例可以考虑术后放、化疗。PPS对单纯放疗或化疗敏感性不高，目前尚无标准化疗方案，多参照非小细胞肺癌。本研究中17例PPS患者不同治疗方式（根治性切除、根治性切除+淋巴结清扫术、根治性切除+淋巴结清扫术+术后放疗或化疗、支气管动脉化疗药物灌注术、部分切除术）的预后差异均未发现统计学差异（*P*=0.89），考虑其原因主要为病例数较少以及预后受肿瘤组织学类型、部位、大小、患者体质等多因素影响，需大量病例其差异才能达到统计学意义。

PPS的预后文献报道不一。有报道^[15]^认为PPS恶性程度较低、病程进展较肺癌缓慢，生存期较肺癌长，5年生存率为45%左右。国内有文献^[18]^报道认为其预后较肺癌差，大部分患者术后2年内多因为远处脏器转移而死亡^[19]^。影响PPS预后的因素有肿瘤的组织学类型、恶性程度、大小、部位、切除是否彻底及有无远处转移^[20]^。PPS的预后分析必须考虑其组织学类型，Janssen等^[2]^报道肺恶性纤维组织细胞瘤、肺滑膜肉瘤、肺平滑肌肉瘤的5年生存率分别为30%、50%和60%。肿瘤直径 > 5 cm者预后不良^[21]^。本研究中预后较文献报道差，可能由于未能早期发现，诊断时肿瘤已较大或已远处转移以及切除不完全所致。

综上，PPS非常少见，单纯根据患者临床症状和影像学表现易误诊，确诊依赖病理学检查尤其是免疫组化。PPS首选手术治疗，可辅以化疗和放疗，预后差，提高对本病的认识，熟悉本病的诊断，尽早手术并完全切除病灶有助于改善预后。
